# Supplementary material for: LIVIA: a browser-based tool for assessing and visualizing predicted protein interactions
Source: bioRxiv. 2026 May 10:2026.05.01.721633. Preprint. [Version 1] doi: 10.64898/2026.05.01.721633 (PMC13174586; doi:10.64898/2026.05.01.721633)
Supplement: Supplement 1 [file NIHPP2026.05.01.721633v1-supplement-1.pdf]

bioRxiv preprint doi: https://doi.org/10.64898/2026.05.01.721633; this version posted May 10, 2026. The copyright holder for this preprint (which was not certified by peer review) is the author/funder, who has granted bioRxiv a license to display the preprint in perpetuity. It is made available under aCC-BY 4.0 International license.

Supplementary Figure

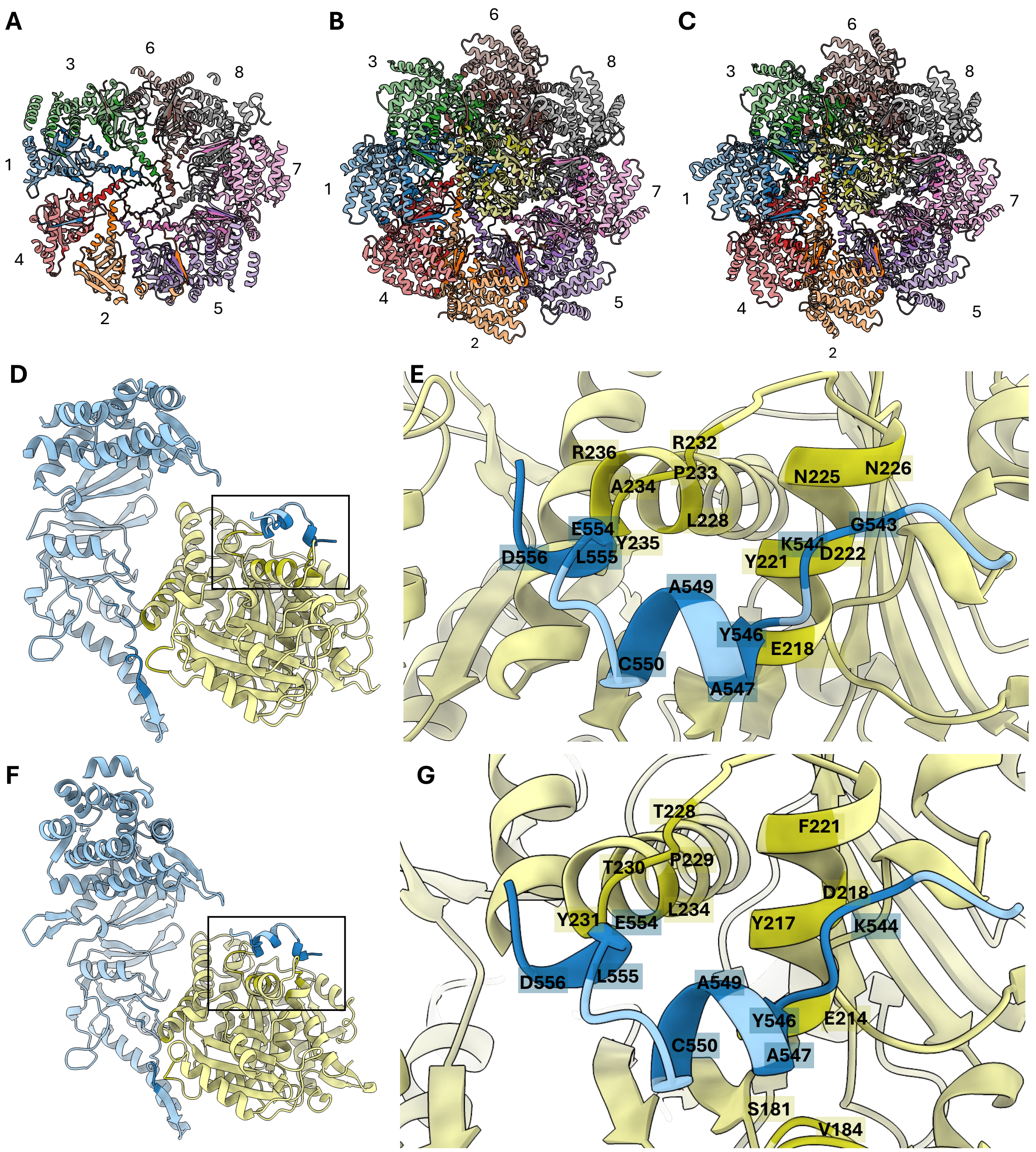

Figure S1. ***Drosophila*** **CCT chaperonin AlphaFold3 predictions in apo form and bound to α- and β-tubulin substrates. (A–C)** ChimeraX views of the full complexes from LIVIA's automatically exported ChimeraX scripts; each subunit is colored individually, with LIR shown in the light chain color and cLIR in the dark chain color. CCT subunits are labeled 1–8 (corresponding to CCT1–CCT8). **(A)** Apo CCT. **(B)** CCT bound to α-tubulin (αTub67C). **(C)** CCT bound to β-tubulin (βTub56D). The substrate-bound complexes show markedly more LIR/cLIR-supported structure than the apo complex (mean pTM 0.58 / ipTM 0.55 for apo vs 0.71 / 0.69 for αTub67C and 0.74

bioRxiv preprint doi: https://doi.org/10.64898/2026.05.01.721633; this version posted May 10, 2026. The copyright holder for this preprint (which was not certified by peer review) is the author/funder, who has granted bioRxiv a license to display the preprint in perpetuity. It is made available under aCC-BY 4.0 International license.

/ 0.73 for βTub56D, across 5 models). **(D, E)** Detailed view of the CCT1–αTub67C pair from the substrate-bound prediction, rendered with LIVIA's automatically exported ChimeraX script. CCT1 is shown in light blue and αTub67C in light olive, with non-interacting regions faded; the box in **D** marks the region magnified in **E**. **(E)** Close-up showing labeled cLIR positions between the CCT1 C-terminal tail and αTub67C. **(F, G)** As in **D, E** but for the CCT1–βTub56D pair. **(F)** Overall dimer view; the box marks the region magnified in **G**. **(G)** Close-up showing labeled cLIR positions between the CCT1 C-terminal tail and βTub56D.
